# Activation of Adenosine A_1_ Receptor in Ischemic Stroke: Neuroprotection by Tetrahydroxy Stilbene Glycoside as an Agonist

**DOI:** 10.3390/antiox10071112

**Published:** 2021-07-12

**Authors:** Lingyu Ruan, Guanghui Li, Wenlong Zhao, Huihui Meng, Qi Zheng, Junsong Wang

**Affiliations:** Center for Molecular Metabolism, Nanjing University of Science and Technology, 200 Xiao Ling Wei Street, Nanjing 210094, China; ruanlingyuamy@njust.edu.cn (L.R.); lguanghui12@njust.edu.cn (G.L.); 217102010089@njust.edu.cn (W.Z.); menghuihui2014@njust.edu.cn (H.M.); zhengqi@njust.edu.cn (Q.Z.)

**Keywords:** neuroprotective agent, adenosine A_1_ receptor agonist, ^1^H-NMR, metabolomics, ERK signal tranduction pathway, ischemic stroke

## Abstract

Ischemic stroke is the main cause of death/disability, posing a great menace to human health. Though efforts to search for therapeutic drugs are ongoing, few of them have succeeded. Adenosine A_1_ receptor (A1R) activation could ameliorate ischemic injury, representing a very tempting target for stroke treatment. Tetrahydroxy stilbene glycoside (TSG), a potent antioxidant from the well-known Chinese herb *Polygonum multiflorum* Thunb., has been reported to have notable neuroprotective activities but the underlying mechanisms are elusive. This study investigated the mechanism of TSG focusing on A1R. TSG markedly decreased mortality, neurological deficit score, cerebral infarct size and brain water content of MCAO rats, and ameliorated the disorders in purine metabolism, energy metabolism and antioxidative defense system. TSG helped the survival of SH-SY5Y cells in OGD/R by alleviating oxidative stress and glutamate release, and by maintaining calcium homeostasis. TSG effects were abolished by A1R antagonist DPCPX. Docking and binding assays confirmed the binding of TSG with A1R. In addition, TSG upregulated the A1R level lowered by MCAO and OGD/R. The downstream signals of A1R activation, ERK1/2, HIF-1α and NF-κB contributed to the neuroprotection of TSG. Moreover, void of “well-known” cardiovascular side effects of classical A1R agonists, TSG showcased its great potential for stroke treatment.

## 1. Introduction

Today stroke is deemed as the second most common cause of death, leading to the most cases of long-term disability worldwide according to the data of the World Health Organization (WHO) [1]. Stroke could be categorized into ischemic and hemorrhagic types, with the former accounting for approximately 80% of all cases. Ischemic stroke is caused by occlusion of a major cerebral artery by a thrombus or an embolism, which leads to block of cerebral blood flow, producing a condition of oxygen and glucose deprivation (OGD). Brain is an important organ with high energy demand, consuming nearly 20% of the whole body energy expenditure. Oxidative phosphorylation of glucose is the major energy supply to sustain the high metabolic activity of the brain. However, the brain has little glycogen and ATP reserves, which made it vulnerable to OGD, and reperfusion can aggravate cerebral damage through ischemia-reperfusion (I/R) injury. Recently, numerous neuroprotective agents targeting ischemia stroke induced-I/R injury have been investigated, and unluckily, few of them have proven efficacious in clinic [2]. While there has significant progress in the area of medical and surgical thrombolytic technologies [3], neuroprotective agents successful at preventing cerebral injury and minimizing disability remain limited. Searching for effective and safe drugs to attenuate and reverse I/R injury will be of great significance.

Under some circumstances, ischemic preconditioning (transient global or focal ischemic attacks) generates neuroprotective events that enhance the brain’s resistance to detrimental effects of subsequent, longer episodes of ischemia [4,5]. Considerable evidence suggested that adenosine release and activation of K_ATP_ channels through adenosine A_1_ receptors (A1R) may constitute an early step in ischemic cerebral preconditioning [6,7]. Activation of A1R by its agonist inhibits adenylyl cyclase via the inhibitory G protein, leading to activation of inwardly rectifying K^+^ channels, and subsequent inhibition of Ca^2+^ channels [8,9]. Inhibition of presynaptic calcium currents decreases release of neurotransmitters, such as glutamate, whose toxicity further aggravates brain damage in ischemic stroke [10,11]. Therefore, A1R agonists are promising in the treatment of cerebral stroke.

*Polygonum multiflorum* Thunb. (PMT) is a famous tonic traditional Chinese medicine that has wide application in clinic to ameliorate learning and memory disorders and treat aging related diseases. In our previous study, the ethanol extract of PMT exerted excellent anti-stroke abilities [12], and other reports have indicated its protective effects against excitatory amino acid toxicity [13] and neuronal degeneration [14]. TSG (tetrahydroxy stilbene glycoside/2,3,5,4’-tetrahydroxystilbene-2-*O*-β-D-glucoside), a resveratrol analog stilbene glucoside of PMT, exerted strong anti-oxidation [15], anti-inflammation [16] and anti-aging effects [17]. Its antioxidative capacity was even higher than resveratrol [18]. TSG showed its protection in a range of experimental models such as Alzheimer’s disease [19], Parkinson’s disease [20], and ischemia [21]. In addition, with potent anti-platelet activity, TSG might also be preventive for persons with high stroke risk [22].

While TSG has been reported to have multifacet effects and to be involved in many pathways, knowledge on the target of TSG for its stroke treatment and neuroprotective effects are scarce [23]. In this study, an I/R model of middle cerebral artery occlusion (MCAO) in SD rats and an OGD/R (OGD/reoxygenation) model in SH-SY5Y cells were used to characterize the neuroprotective effects of TSG both in vivo and in vitro. Target prediction, molecular docking, drug affinity responsive target stability (DARTS) assay and ^1^H-NMR based metabolomics analysis gave rich information and clues on its target underlying the anti-stroke effects of TSG. In this study, we found that TSG activated A1R as an agonist and then mediated several downstream signaling pathways, thus protecting cells from damage arising from energy metabolism disorder and oxidative stress during I/R.

## 2. Materials and Methods

### 2.1. Chemicals and Reagents

**Figure 1:**
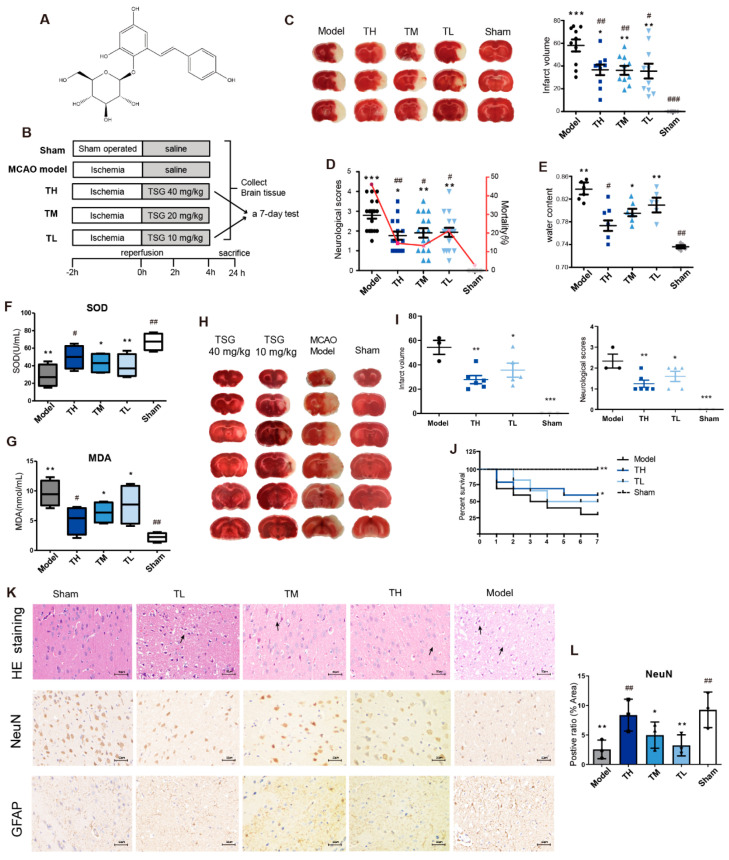
Protection of TSG on cerebral I/R injury in the MCAO rats. (**A**) The structure of TSG. (**B**) Experimental procedure of testing 24 h/7 day TSG efficacy effects in the MCAO rats. (**C**) TTC staining of cerebral slices and cerebral infarct volume (% of hemisphere), *n* = 10. (**D**,**E**) Neurobehavioral scores, mortality of rats and brain water content (*n* = 6). (**F**,**G**) Boxplots of biological parameters SOD, MDA (*n* = 4). (**H**,**I**) TTC staining of cerebral slices, infarct volume and neurobehavioral scores of MCAO rats after a 7 day TSG treatment. (**J**) Kaplan–Meier survival curve of rats in 7 days. (**K**,**L**) HE staining, immunohistochemistry of NeuN and GFAP of brain sections. Positive ratio (% of area) of NeuN immunostaining (*n* = 3). Black arrows indicated perineuronal vacuolization of the infarcted cortex region. Scale bar represents 50 µm. # *p* < 0.05, ## *p* < 0.01 and ### *p* < 0.001, other groups vs. model group; * *p* < 0.05, ** *p* < 0.01 and *** *p* < 0.001, other groups vs. sham group.

TSG (CAS: 82373-94-2; purity: 99.42%; structure as shown in Figure 1A) was purchased from Yuanbaofeng Medical Technology Co, Ltd. (Nanjing, China). Silicone-coated monofilament nylon suture was purchased from Guangzhou Jialing Biotechnology Co, Ltd. (Guangzhou, China); 3-trimethyl-siloxane-sodium propionate (TSP), 8-Cyclopentyl-1,3-dipropylxanthine (DPCPX) and nimodipine was purchased from Sigma-Aldrich (St. Louis, MO, USA). Deuterium oxide (D_2_O, 99.9%) was purchased from Sea Sky Bio Technology Co, Ltd. (Beijing, China).

For cell cultures, such as Opti-MEM, DMEM and RPMI 1640 culture medium, lipofectamine 2000 reagent, fetal bovine serum (FBS), sodium pyruvate, Glutamax, NEAA, penicillin (100 U/mL) and streptomycin (0.1 mg/mL) were obtained from Gibco Invitrogen (Carlsbad, CA, USA). Protease inhibitor, CCK-8 Reagent, BeyoGold™ His-tag Purification Resin, Fura-2/AM, nuclear/membrane and cytoplasmic protein extraction kits, fluorescent probe 2′,7′-dichlorofluorescein diacetate (DCFH-DA), proteinase K and protease inhibitor cocktail were purchased from Beyotime Biotechnology Co., Ltd. (Shanghai, China). SYBR Green PCR Master Mix was obtained from SCIEX (Foster City, CA, USA). The primary antibodies adenosine A_1_ receptor (A1R, catalog number: 55026-1-AP), nuclear factor-kappa B (NF-κB, 66535-1-Ig) and β-actin (66009-1-Ig) were bought from Proteintech Group, Inc. (Chicago, IL, USA). Extracellular signal-regulated kinases-ERK1/2 (Thr202/Tyr204, catalog number: AF0155), pERK1/2 (AF1015) were purchased from Affinity BioReagents (Golden, CO, USA). Transcription factor hypoxia-inducible factor-1 (HIF-1α, catalog number: 14179), HRP-conjugated rabbit anti-mouse secondary antibody IgG and goat anti-rabbit secondary antibody IgG were purchased from Cell Signaling Technology (Danvers, MA, USA).

The inhalational anesthetic isoflurane was purchased from RWD Life Science (Shenzhen, China). Triphenyl-2,3,4-tetrazolium-chloride (TTC) were obtained from Wako Pure Chemical Industries (Osaka, Japan). The reagent kit for determining lactate dehydrogenase (LDH), reactive oxygen species (ROS), superoxide dismutase (SOD), malondialdehyde (MDA) and glutamate were purchased from Nanjing Jiancheng Institute of Biological Engineering (Nanjing, China). TRIzol RNAiso Plus reagent was bought from TaKaRa (Dalian, China). Reverse transcript enzyme kits were obtained from KeyGen Biotech (Nanjing, China). Enhanced chemiluminescence ECL kit was purchased from Santa Cruz Biotechnology, Inc. (Santa Cruz, CA, USA). Neuronal nuclear antigen (NeuN) and glial fibrillary acidic protein (GAFP) antibodies were obtained from Abcam (Cambridge, UK). Geneticin (G418) and bicinchoninic acid (BCA) Protein Assay Kit were bought from Solarbio Life Science (Beijing, China).

### 2.2. MCAO Model Establishment and Drug Administration

Adult male Sprague–Dawley (SD) rats (230–250 g) were purchased from Yangzhou University (Yangzhou, China). Rats were reared under standard housing conditions at 22 °C, with a 12 h light–dark cycle and were given food and water ad libitum for acclimation. All procedures were approved by the Animal Ethics Committee of Nanjing University of Science & Technology (Ethics Approval ID number: ACUC-NUST-20180039), and carried out in accordance with the National Institutes of Health Guidelines for Animal Research.

MCAO operation was performed as reported previously [24]. Briefly, rats were fasted overnight and then induced inhalation anesthesia with 5% isoflurane and maintained by 1.0–1.5% isoflurane using an anesthesia machine (EZVET F710, RWD Life Science). The body temperatures were maintained at 37.0 °C with warming pads. A ventral midline incision was made at the neck area, and the sternohyoid and sternomastoid muscles were bluntly dissected to expose the common carotid artery (CCA). The right sides of the external carotid artery (ECA) and internal carotid artery (ICA) were isolated and mobilized (freed) from the surrounding tissues very near the skull base. A small incision was made in the ECA and a silicone-coated monofilament nylon suture (Guangzhou Jia Ling Biotechnology Co, Ltd., Guangzhou, China) was gently inserted into the ICA through the ECA stump within about 18 to 20 mm from the fork. Monofilaments could be prepared differently according to a specific study regimen (monofilament diameter is dependent on rat weight). One part of the filament, ca 5- to 7-mm length, remained outside and was withdrawn after 120 min to allow reperfusion. Rats were re-anesthetized with isoflurane to relieve pain during the withdrawal. Sham-operated rats underwent the same procedure but without arterial occlusion.

Rats after MCAO surgery were randomly assigned to different treatments into four groups: MCAO model group and three TSG groups, TL, TM and TH corresponding to low-, medium- and high-doses with dosages of 10, 20 and 40 mg/kg (*n* > 15/group). TSG of different doses (dissolved and diluted in saline) was subcutaneously (s.c.) injected to the rat’s neck at 0, 2 and 4 h after reperfusion (Figure 1B). The sham and model groups were given the same volume of physiological saline. Some rats were excluded because of surgical failure, death resulted from severe cerebral infarction within 24 h, treatment failure and other abnormalities. For all animals, before sacrifice, the neurological function was assessed by two investigators who were blinded to the experimental design. Neurological deficits were quantified as described previously using a five-point scale: 0, normal, no observable deficits; 1, slight, fail to extend the left forepaw; 2, moderate, circling to the left; 3, severe, lean to the left; and 4, serious, unconsciousness and fail to move autonomously [24]. Data points were included only when the difference of scores given by the two evaluators on one sample was no greater than 1 point. The excluded rat brains were tested by TTC staining for their infarction status. Rats that survived 24 h after surgery were sacrificed and then brain samples were collected and stored at −80 °C.

Thirty MCAO rats scored 2–3 were s.c. administered with TSG for 7 days (Model, TSG 10, and 40 mg/kg, twice a day, ten rats each group) to investigate its neuroprotective effects in a longer treatment window. Rats in the model group were given the same volume of physiological saline. To test whether A1R was involved in TSG anti-stroke activity, DPCPX, an A1R antagonist, was applied to MCAO rats in advance. DPCPX (0.25 mg/kg, s.c.) was pretreated before operation, followed by TSG (40 mg/kg, s.c.) treatment at ischemia, reperfusion 0 and 4 h. Other procedures in these MCAO experiments were the same as in Section 2.2 described above.

### 2.3. TTC, HE Staining and Immunohistochemistry

After MCAO, the rats were anesthetized with isoflurane (EZVET F710, RWD Life Science) and sacrificed. The brains of rats were removed and cut into 2-mm-thick coronal sections for TTC staining. The brain sections (*n* = 10/group) were immersed in 2% TTC (dissolved in saline) at 37 °C for 30 min. The infarct areas of all sections from one sample were photographed and quantified using ImageJ (http://rsb.info.nih.gov/ij/, NIH, USA, accessed on 10 December 2018) to calculate the infarct volume (% hemisphere). For hematoxylin and eosin (HE) staining, fresh brains were immediately immersed in 10% phosphate-buffered formalin for 12 h fixation, and then embedded in paraffin. Adjacent brain sections (5 µm thick) were cut in the coronal plane and then stained with H&E to examine the histopathological changes.

For immunohistochemistry, brain tissues (*n* = 3/group) were placed in 1–4 °C normal saline and cut into 5 µm frozen sections. Next, the sections were treated with 3% H_2_O_2_ for 30 min to block endogenous peroxidase activity, then incubated in 3% BSA at 37 °C for 30 min and subsequently incubated with GFAP/NeuN antibody in a wet box at 4 °C overnight. After incubation, the sections were washed three times with PBST (phosphate-buffered saline containing 0.05% Tween-20) and then incubated with corresponding secondary antibody at 37 °C for 1 h. Lastly, the sections were stained with diaminobenzidine (DAB) at room temperature for 10 min, and hematoxylin for 3 min. A brown signal indicates positive staining by DAB and a blue signal indicates nuclear counterstaining by hematoxylin. Three randomly selected fields around the cerebral cortex penumbra in NeuN immunostained sections were photographed and analyzed in ImageJ to obtain positive cell ratios (% area).

### 2.4. Sample Preparation, Acquisition of ^1^H NMR Spectra and Data Processing

Frozen ischemic hemispheres were homogenized in ice-cold solvent (50% acetonitrile, 5 mL/g tissue), vortexed and centrifuged at 12,000× *g* for 10 min at 4 °C. The supernatant was collected and concentrated under a stream of nitrogen and lyophilized. Dried cerebrum extract was reconstituted with 0.5 mL D_2_O phosphate buffer (0.2 mol/L Na_2_HPO_4_ and 0.2 mol/L NaH_2_PO_4_, pH 7.4, containing 0.05% TSP). After vortex to fully dissolve the powder and centrifugation (12,000× *g*, 10 min, 4 °C) to remove any residues, the transparent supernatant solution was pipetted into 5 mm NMR tubes for NMR analysis.

**Figure 2:**
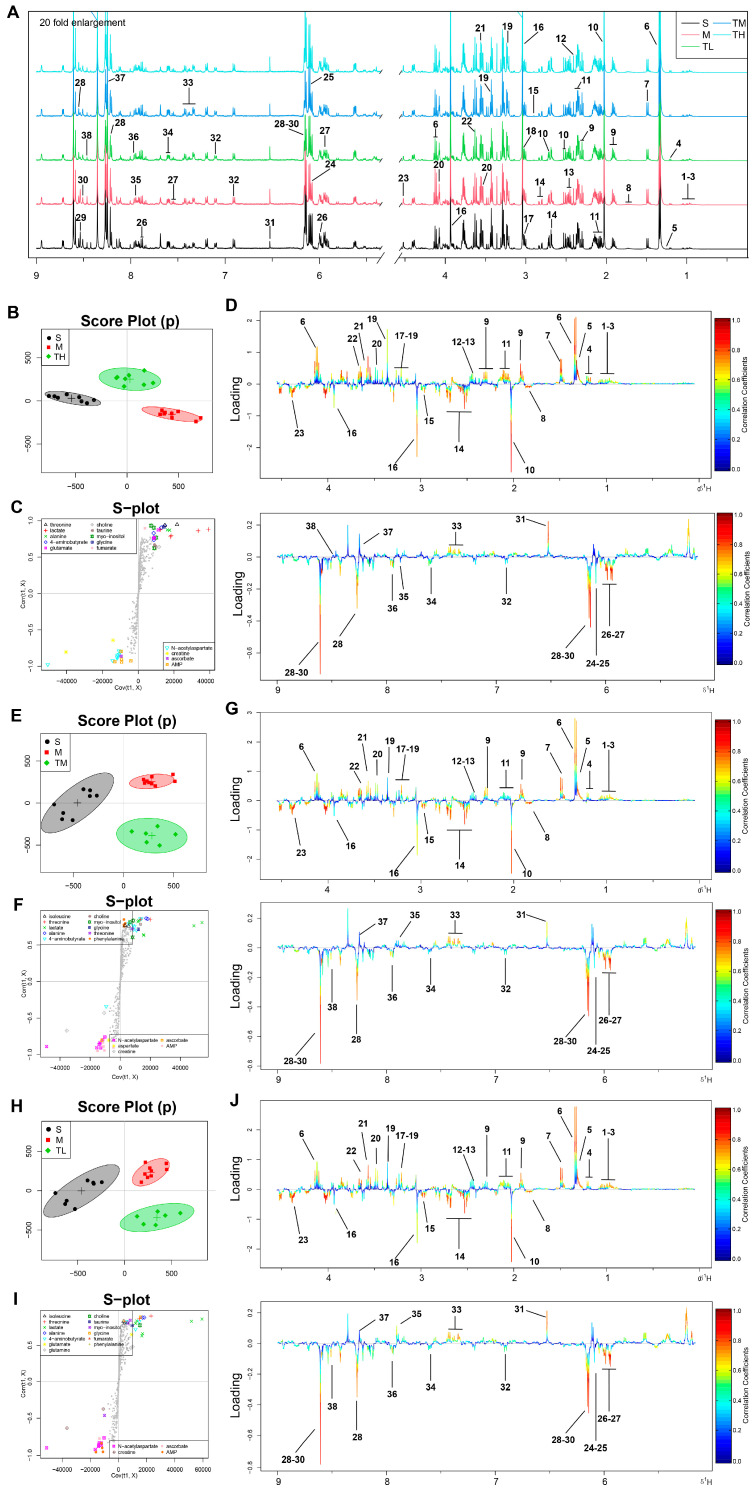
OSC-PLSDA analysis of the brain metabolic profiles between sham, model and TH/TM/TL groups. (**A**) Typical 500 MHz ^1^H NMR spectra of brain extracts with the metabolites labeled for all groups. Metabolites: 1, isoleucine; 2, leucine; 3, valine; 4, 3-hydroxybutyrate; 5, 2-hydroxyisobutyrate; 6, lactate; 7, alanine; 8, lysine; 9, 4-aminobutyrate; 10, N-acetyl aspartate; 11, glutamate; 12, succinate; 13, glutamine; 14, aspartate; 15, trimethylamine; 16, creatine; 17, choline; 18, phosphocholine; 19, taurine; 20, myo-inositol; 21, glycine; 22, threonine; 23, ascorbate; 24, adenosine; 25, inosine; 26, uridine; 27, uracil; 28, AMP; 29, ADP; 30, ATP; 31, fumarate; 32, tyrosine; 33, phenylalanine; 34, niacinamide; 35, histidine; 36, xanthine; 37, oxypurinol; 38, formate; consistent with Supplementary Table S1. (**B**,**E**,**H**) OSC-PLSDA score plots. (**C**,**F**,**I**) OSC-PLSDA S-plots. (**D**,**G**,**J**) Color-coded coefficient loading plots. Blue: Lowest, no statistically significant difference between the groups; red: Highest, statistically significant. Metabolites are numbered consistently with those in Figure 2A.

NMR measurements were carried out at 25 °C on a Bruker AVANCE 500 MHz system (Bruker BioSpin GmbH, Rheinstetten, Germany). For each sample, the transverse relaxation-edited Carr–Purcell–Meiboom–Gill (CPMG) spin-echo pulse sequence (RD-90°-(τ-180°-τ) n-ACQ) was used with a total spin-echo delay (2 nτ) of 40 ms. Then, 128 free induction decays (FIDs) were collected into 32K data points using a spectral width of 12 ppm with an acquisition time of 3.27 s and a relaxation delay of 3.0 s. The one-dimensional (1D) spectra were manually phased, baseline corrected and referenced to TSP (CH3, δ 0.00) using the Bruker Topspin3.0 software (Bruker GmbH, Karlsruhe, Germany). The ASCII files of NMR spectra obtained accordingly were binned and normalized for multivariate data analysis with an in-house developed R-script (http://cran.r-project.org/, accessed on 10 October 2018). Brain metabolites were identified with the aid of Chenomx NMR suite software (Version 8.1, Chenomx, Inc., Edmonton, Canada) by referencing reported data and querying publicly accessible metabolomics databases (HMDB, http://www.hmdb.ca/, accessed on 11 December 2018; MMCD, http://mmcd.nmrfam.wisc.edu/, accessed on 11 December 2018) detailed in Figure 2A and Supplementary Table S1.

### 2.5. Metabolomic Analysis and Correlation Network Analysis

A supervised orthogonal signal correction partial least squares discriminant analysis (OSC-PLSDA) was performed for multivariate statistical analysis to maximize the discrimination between classes by filtering irrelevant effects [25]. Metabolic patterns between groups were shown in the score plots; differential metabolites were visualized by S-plots and loadings plots color coded with the absolute value of coefficients (Figure 2B–J). The fold-change values of metabolites and the associated *p*-values corrected with the Benjamini and Hochberg (HB) method [26] are listed as a colored table (Table 1).

**Figure 3:**
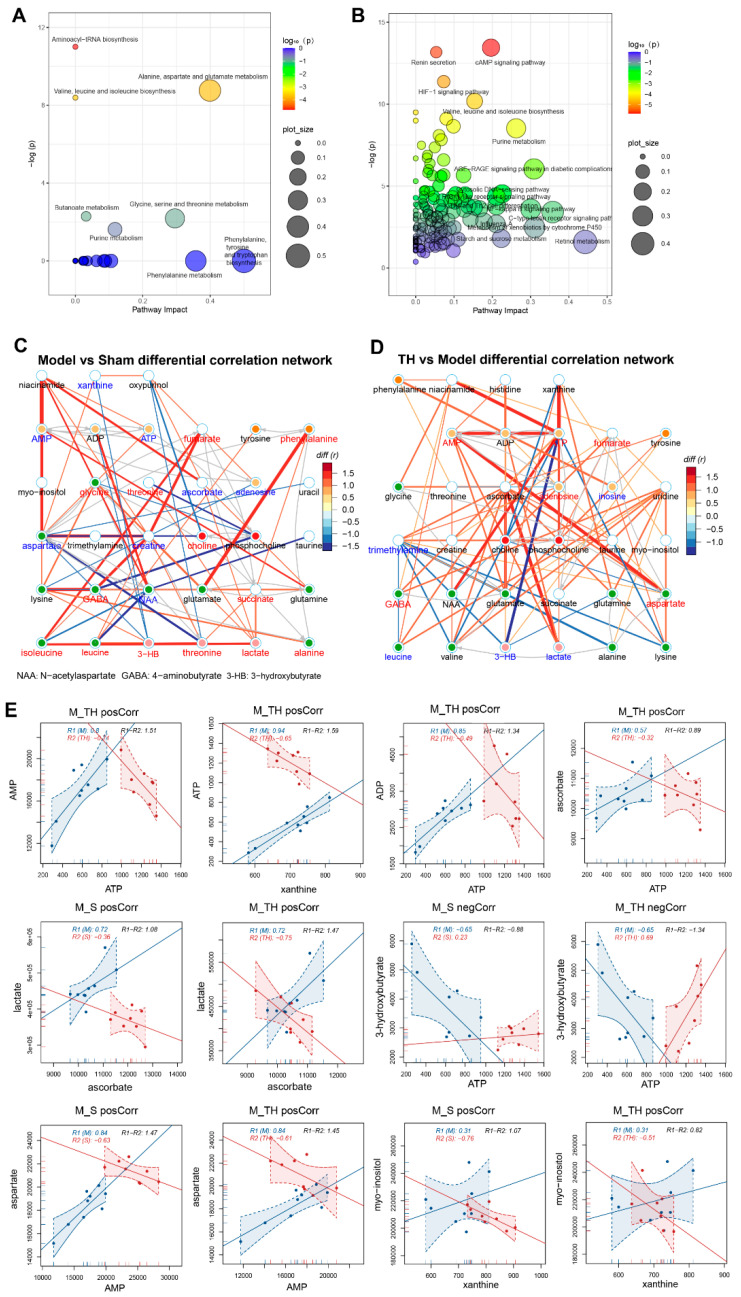
Metabolomics pathway and correlation network analysis of TSG putative targets. (**A**,**B**) Impact of pathways enriched on the disturbed metabolites and predicted targets of TSG in bubble plots. The color of each circle is based on *p*-values (darker colors indicate more significant changes of metabolites in the corresponding pathway), whereas the size of the circle corresponds to the pathway impact score. The most impacted pathways with high statistical significance scores are annotated. (**C**,**D**) Differential correlation network analysis of brain metabolic profiles between the sham, model and TH groups. Metabolites with Pearson’s correlations coefficients over 0.65 and *p*-values less than 0.05 are connected by solid lines that are color coded according to the values of the coefficients; a warm color represents a positive correlation, and a cool color represents a negative correlation. The width of each line was scaled based on its absolute values. The names of the metabolites shown in red and blue indicated that they are significantly increased and decreased, respectively. The gray lines between the metabolites indicated direct biological reactions. (**E**) Scatter plots of correlated metabolites. Each point represents one sample. R1 and R2 indicate the Pearson coefficient between two metabolites in each two group.

Differential networks between groups were constructed using R package igraph. Metabolite pairs with differential Pearson’s correlation coefficients over 0.65 were linked with lines color and width coded according to the absolute correlation coefficients: Reds and blues indicated positive and negative correlations, respectively; the thicker the line, the bigger the absolute values of the differential correlation coefficients. Metabolites with similar structures (Tanimoto index over 0.7) were filled with the same color and connected by gray lines (Figure 3C,D). The differential correlation networks were further mapped to the biochemical reaction networks by connecting metabolites with gray arrows. Detailed correlations between two metabolites are shown in scatter plots (Figure 3E).

### 2.6. Target Prediction and Pathway Enrichment Analysis

Potential targets of TSG were fished and collated in Supplementary Table S2 with a Swiss Target Prediction web server (http://www.swisstargetprediction.ch/index.php, accessed on 24 February 2019) [27]. Pathway enrichment analysis was performed in MetaboAnalyst (http://www.metaboanalyst.ca/, accessed on 13 March 2019) with our metabolomics data and predicted targets [28]. In the bubble plots, each circle represents a different pathway whose size and color are based on its impact and significance (red being the most significant), respectively (Figure 3A,B).

### 2.7. Molecular Docking Experiments

AutoDock4 (version 4.2.6) and AutoDock Vina software are used for molecular docking studies in our study [29,30]. The structure of A1R (PDB code: 6D9H) was downloaded from UniProt (https://www.uniprot.org/, accessed on 6 March 2020). The grid box size was set at 40, 40 and 40 Å for x, y and z, respectively. The spacing between the grid points was 1.0 Å. The grid center was set at 92.16, 120.297 and 92.496 Å for x, y and z, respectively. The Lamarckian Genetic Algorithm (LGA) was chosen to search for the best conformers. During the docking process, a maximum of 10 conformers was considered for each ligand. All the docking processes were performed with the default parameters of AutoDock4 and AutoDock Vina running under the Windows 10 Operating System. All figures with structure representations were produced by PyMOL and Discovery Studio Visualizer (Figure 5).

### 2.8. Constructiontion of HEK293T Cell Lines Stably Expressing Human A1R

HEK293T cells were kindly provided by Stem Cell Bank, Chinese Academy of Sciences. HEK293T cells (passaged within six passages) were cultured in a DMEM culture medium containing 1% penicillin/streptomycin and 10% FBS at 37 °C in 5% CO_2_. A HEK293T-derived cell line stably expressing human A1R clones was established by transfection with a pcDNA3.1(+) plasmid encoding C-terminal 6-His-tagged (C-His) A1R using Lipofectamine 2000 reagent (GIBCO, Invitrogen, Carlsbad, CA, USA). The transfected HEK293T/A1R cells were selected with 600 µg/mL G418 geneticin (Solarbio, Beijing, China) in a DMEM medium for three weeks [31,32]. Between 2 and 3 weeks into the selection process, G418 resistant cells began to appear. Stably transfected HEK293T/A1R cell lines were sought out by serial dilutions and allowed to grow from single cells in a DMEM high-glucose medium containing 1% penicillin/streptomycin, 300 µg/mL G-418, and 10% FBS.

The coding sequence (CDS) of human A1R was obtained from NCBI (https://www.ncbi.nlm.nih.gov/, accessed on 1 March 2019): atgccgccctccatctcagctttccaggccgcctacatcggcatcgaggtgctcatcgccctggtctctgtgcccgggaacgtgctggtgatctgggcggtgaaggtgaaccaggcgctgcgggatgccaccttctgcttcatcgtgtcgctggcggtggctgatgtggccgtgggtgccctggtcatccccctcgccatcctcatcaacattgggccacagacctacttccacacctgcctcatggttgcctgtccggtcctcatcctcacccagagctccatcctggccctgctggcaattgcggtggaccgctacctccgggtcaagatccctctccggtacaagatggtggtgaccccccggagggcggcggtggccatagccggctgctggatcctctccttcgtggtgggactgacccctatgtttggctggaacaatctgagtgcggtggagcgggcctgggcagccaacggcagcatgggggagcccgtgatcaagtgcgagttcgagaaggtcatcagcatggagtacatggtctacttcaacttctttgtgtgggtgctccccccgcttctcctcatggtcctcatctacctggaggtcttctacctaatccgcaagcagctcaacaagaaggtgtcggcctcctccggcgacccgcagaagtactatgggaaggagctgaagatcgccaagtcgctggccctcatcctcttcctctttgccctcagctggctgcctttgcacatcctcaactgcatcaccctcttctgcccgtcctgccacaagcccagcatccttacctacattgccatcttcctcacgcacggcaactcggccatgaaccccattgtctatgccttccgcatccagaagttccgcgtcaccttccttaagatttggaatgaccatttccgctgccagcctgcacctcccattgacgaggatctcccagaagagaggcctgatgactag (Gene ID Accession Numbers: S45235). The plasmid with C-His A1R was synthesized by GenScript Biotech Corporation, Nanjing China.

### 2.9. DARTS Assay

In order to identify the binding of TSG/DPCPX with A1R, the DARTS approach was used (Figure 6A) based on the fact that protein-ligand binding could increase the stabilization of the protein against protease digestion [33]. Proteins of fresh rat cerebral cortex were extracted with membrane and cytoplasmic protein extraction kits (Beyotime, Shanghai, China), lysed in 20% w/v of lysis buffer, then centrifuged at 13,300 rpm (10 min; 4 °C) to afford the suspension for protein quantitation and binding assay. Following a 30 min incubation at 37 °C with TSG (10, 100 µM)/DPCPX (5 µM), the suspension (15 µg/µL protein) was digested with proteinase K diluted (1:1000) in TNC buffer (50 mM Tris-HCl, pH 8.0, 50 mM NaCl, 10 mM CaCl_2_) for 30 min at 37 °C. The digestion was stopped by heating with 5× SDS-PAGE loading buffer. The reaction results were analyzed by SDS-PAGE followed by Coomassie Brilliant Blue G-250 staining.

Transiently transfected HEK293T/A1R cells were added with 100 µL non-denatured lysis buffer (protease inhibitors and phosphatase inhibitors added in advance) per dish and lysed by repeated freeze-thaw cycles. The protein mixture was centrifuged at 15,000× *g* (10 min; 4 °C) to afford suspension (100 µL), and incubated with 20 µL 50% BeyoGold^TM^ His-tag Purification Resin (Beyotime, Shanghai, China) on a shaker overnight at 4 °C. After washing and centrifugation cycles (3 × 10 s, 1000× *g*, 4 °C), the pellets were gently resuspended in lysis buffer containing non-denaturing detergent (Beyotime, Shanghai, China), then centrifuged at 1000× *g* (10 s; 4 °C) to afford suspension for A1R protein purification. The protein concentration was determined using the BCA Protein Assay Kit (Solarbio, Beijing, China).

### 2.10. OGD/R Model

Human neuroblastoma SH-SY5Y cells (a neuroblastoma cell line, provided by Stem Cell Bank, Chinese Academy of Sciences, Beijing, China) were cultured in 1:1 mixture of MEM and F12 medium, supplemented with 1% penicillin/streptomycin, 1% sodium pyruvate, 1% Gluta-max, 1% NEAA and 10% FBS. Cells (1 × 10^5^) passaged for fewer than six passages were seeded into a cell culture plate 36 h before OGD/R experiments. Cells were rinsed with phosphate-buffered saline (PBS) three times and incubated in a glucose- and FBS-free medium. Then, cells were immediately placed in the HF100 tri-gas incubator (Heal Force Bio-Meditech Holdings Limited, Shanghai, China) pre-flushed with a 5% CO_2_/95% N_2_ gas mixture to form an anoxic chamber. With an oxygen detector (AS8801, Smart Sensor, Guangzhou, China), the O_2_ concentration was monitored and maintained at 0.1%. After 3 h OGD, cells were returned to the normoxic incubator with normal culture medium and incubated for another 24 h before harvest.

TSG was dissolved in DMSO and subsequently diluted with the culture medium (final DMSO concentration for experiments was lower than 0.1%) to different concentrations. A dose-response test of TSG was carried out in SH-SY5Y cells to obtain the best working concentrations (Figure S1A). Then cells were divided into the following groups: CTSG/control group: Cells with/without TSG (4/8 µM) treatment; OGD/R group: Cells subjected to OGD/R; OGD group: Cells subjected to OGD without reperfusion; TSG (4/8 µM) treatment groups: Cells incubated with TSG of different dose during OGD/R; DPCPX (5 µM) treatment group: Cells incubated with DPCPX during OGD/R; DPCPX (5 µM) + TSG (8 µM) treatment group: Cells treated with DPCPX right before TSG treatment and incubated with this two-drug combination during OGD/R. To examine OGD/R-induced cytotoxicity and the effect of TSG against OGD/R injury, cell viability was examined by CCK-8 assay following the manufacturer’s instructions.

### 2.11. Biochemical Indexes Assays

Intracellular reactive oxygen species (ROS) formation was measured by fluorescent probe 2′,7′-dichlorofluorescein diacetate (DCFH-DA, Beyotime). After OGD/R, cells were washed twice with PBS, and then incubated with 10 µmol/L DCFH-DA for 30 min at 37 °C. Fluorescence intensity was observed in a fluorescence microscope (Nikon 80i; Tokyo, Japan) at excitation and emission settings of 488 nm and 525 nm. Intracellular Ca^2+^ concentration changes were measured using the fluorescent probe Fura-2 AM (Molecular Probes, Beyotime). After OGD, cells were incubated with 5 µM of Fura-2 AM for 45 min in the dark at 37 °C. Cells were washed twice with HBSS buffer (143 mM NaCl, 5.6 mM KCl, 0.34 mM Na_2_HPO_4_, 0.44 mM KH_2_PO_4_, 0.42 mM NaHCO_3_, 5.6 mM glucose, 10 mM HEPES, 2 mM CaCl_2_, 0.8 mM MgCl_2_, adjusted to pH 7.2) and excited at 340 nm and imaged at 510 nm in Nikon 80i fluorescence microscope. The mean fluorescent intensity was calculated by ImageJ software. Extracellular LDH and glutamate in cell culture medium were measured by commercial assay kits according to the instructions. The level of oxidative stress in the brain of MCAO rats was determined by measuring the SOD activity and MDA levels using commercial assay kits according to the manufacturer’s instructions.

### 2.12. Western Blotting (WB) and Q-RT-PCR

The proteins for WB were extracted from the frozen brain tissues, HEK293T cells, HEK293T/A1R cells and SH-SY5Y cells with protein extraction kits according to the instructions. Protein concentrations were determined using the BCA assay (Solarbio, Beijing, China). Protein samples were fractionated on 10% SDS-PAGE and transferred onto nitrocellulose membranes. Membranes were then blocked with 5% skim milk which soluble in TBST (Tris-buffered saline with 0.1% Tween-20, pH 7.6) at room temperature for 2 h, and incubated overnight at 4 °C with primary antibodies (A1R, NF-κB, β-actin, ERK1/2, pERK1/2). The bound primary antibodies were labeled with corresponding secondary antibodies (goat anti-rabbit/mouse IgG; diluted 1:5,000 in 5% skim milk) for 1 h at room temperature. The bound antibodies were visualized using ECL kits (Santa Cruz, CA, USA) and then images were acquired with a ChemiScope 6100 imaging system (CliNX Science Instruments, Shanghai, China).

The total RNA of brain was extracted from the TRIzol homogenate according to the manufacturer’s instructions. The frozen brain was ground in liquid nitrogen and homogenized in TRIzol reagent (TaKaRa, Dalian, China). RNA concentration was calculated from the optical density at 260 nm, and purity was determined by 260 nm/280 nm absorbance with a Nanodrop Spectrophotometer (BioDrop, Cambridge, UK). Next, reverse transcription reaction was carried out to obtain cDNAs with Reverse Transcription System (KeyGen, Nanjing, China). Quantitative real-time PCR was carried out on an ABI 7300 Real-Time PCR system (SCIEX, Foster City, CA, USA) with SYBR Green PCR Master Mix (SCIEX, Foster City, CA, USA) according to the manufacturer’s instructions. The primers were: ADORA1: Forward 5′-AAC CTG AGT GTG GTA GAG CAA GAC-3′ and reverse 5′-AGT CCT CAG CTT TCT CCT CTG GG-3′ (Accession: XM_039090620.1); β-actin: Forward 5′-ACA CTG TGC CCA TCT ACG AGG-3′ and reverse 5′-AGG GGC CGG ACT CGT CAT ACT-3′ (Accession: XM_040455798.1). Three replicates for each experimental sample were performed. A1R expression levels normalized to β-actin were calculated by comparative threshold cycle method (2-DeltaDeltaCt).

### 2.13. Determination of Blood Pressure and Heart Rate

A noninvasive computerized tail-cuff system (CODA Monitor System, Kent Scientific Corporation, Torrington, CT, USA) was applied to determine the blood pressure and heart rate of conscious rats. Rats were randomly divided into four groups: Control group (Con), and TL, TM and TH groups corresponding to low, medium and high doses with dosages of 10, 20 and 40 mg/kg. The blood pressure and heart rate of rats in each group were measured and recorded for 14 days before TSG treatment. Starting on the 15th day, TSG of different doses was subcutaneously (s.c.) injected into rats twice a day for 21 days. Rats in the sham group were injected with saline. Blood pressure and heart rate were recorded every day throughout the administration.

### 2.14. Data Analysis

All data were expressed as mean ± SD analyzed by GraphPad Prism 4.0 software (GraphPad Software, San Diego, CA, USA). One-way ANOVA with Newman–Keuls posthoc test was used to evaluate the significance of all pairs. The following terminology is used to denote the statistical significance: # *p* < 0.05, ## *p* < 0.01 and ### *p* < 0.001 vs. model, OGD or OGD/R groups, * *p* < 0.05, ** *p* < 0.01 and *** *p* < 0.001 vs. sham or control groups.

## 3. Results

### 3.1. TSG Ameliorated Cerebral I/R Injury in MCAO Rats

The cerebral infarct volume was 58.07 ± 5.314% in MCAO rats, which was significantly decreased by TSG to 36.63 ± 4.578% (TH), 36.23% ± 3.954% (TM) and 35.52% ± 6.513% (TL) (Figure 1C). As compared with the model group (44.4%), the mortality was significantly decreased in three TSG groups, TH (14.28%), TM (12.5%) and TL (21.42%) in Figure 1D. TSG greatly improved neurobehavior of MCAO rats. The mean neurobehavioral deficit score was decreased from 2.8 in the model group to 1.77, 1.90 and 1.93 in the TH, TM and TL groups, respectively. TSG treatment significantly decreased water content induced by MCAO in a dose-dependent manner (Figure 1E). TSG exhibited marked antioxidant effects (Figure 1F,G), significantly decreased levels of lipid peroxidation product MDA and increased activities of anti-oxidase SOD in MCAO rats. To investigate the benefits of TSG on stroke recovery, another experiment with seven days of consecutive TSG treatment after modeling was performed. TSG treatment significantly increased the survival of MCAO rats and substantially decreased both neurological score and total infarction volume (Figure 1H–J), showcasing its wide therapeutic time window.

### 3.2. TSG Administration Relieved Brain Cell Injury Induced by MCAO

In Figure 1K, darkly stained pyknotic nuclei, cell body shrinkage and perineuronal vacuolization (black arrows) of the infarcted cortex region in the model group were revealed by microscopic analysis. TSG treatment notably decreased the number of darkly stained nuclei; thus, the neuropil was less vacuolized in the infarcted cortex region of the TL, TM and TH groups. Immunohistochemical staining with the anti-NeuN and anti-GFAP antibody was used to detect intact neurons and injured glial cell activation in brains of MCAO rats. NeuN immunostaining revealed that neuronal degeneration occurred in the MCAO model group, which was in consistent with what observed in the H&E staining. The expression ratio of NeuN was greatly decreased in the model group (Figure 1L), reflecting severe neuronal damage or death, which was dose-dependently ameliorated by TSG treatment. GFAP immunostaining showed the typical staining of stellate, branched astrocytes in the sham group. The penumbra of the cortex in the MCAO model group showed increased GFAP immunoreactivity and hypertrophied morphology. Impaired glial cell activation markedly increased infarct size and apoptotic neurons following ischemia. TSG treatment protected glial cells in a dose-dependent manner as evidenced by a significant decrease of GFAP immunoreactivity, especially in the TH group.

### 3.3. TSG Ameliorated the Disturbed Metabolic Profiles of MCAO Rats towards Normal Status

Typical ^1^H NMR spectra for brain extracts from five groups of rats were presented in Figure 2A with a total of 38 metabolites denoted, whose assignments were detailed in Supplementary Table S1. The corresponding fold changes and *p*-values were presented in Table 1. The representative score plots, S-plots and color-coded loading plots of the sham (S), model (M) and TH groups are presented in Figure 2B–D, respectively; those of the sham (S), model (M) and TM/TL groups are presented in Figure 2E–J. The sham and model groups were furthest away in the OSC-PLSDA score plots with the TH (high dosed TSG) group in between, demonstrating significant metabolic disturbances in the brain of MCAO rats, which could be greatly ameliorated by high-dose TSG. In the S-plots and loading plots, lactate, alanine, leucine, isoleucine, valine, 3-hydroxybutyrate, 2-hydroxyisobutyrate, glycine, fumarate, threonine, phenylalanine, formate and choline were markedly increased in the brain after MCAO, while AMP, N-acetyl-aspartate (NAA), ATP, adenosine, ascorbate, creatine, aspartate, 4-aminobutyrate (GABA) and xanthine were significantly decreased. High-dose TSG could remarkedly reverse the changes of key disturbed metabolites, such as ATP, AMP, ADP, lactate, adenosine and 3-hydroxybutyrate in the brain.

### 3.4. Pathway Enrichment Analysis of TSG Affected Metabolites and Predicted Targets

To have a better understanding of the altered metabolites, MetaboAnalyst was used to study the overview of systematic metabolome changes based on pathway analysis. The most relevant pathways on the basis of metabolites were amino acid (valine, isoleucine, leucine, alanine, aspartate) metabolism, purine metabolism and butanoate metabolism (Figure 3A). The predicted targets of TSG (Table S2) were further incorporated to reveal more related pathways, which revealed great impact of cAMP signaling pathway, HIF-1 signaling pathway, valine, leucine and isoleucine biosynthesis, purine metabolism and AGE-RAGE signaling pathway (Figure 3B).

### 3.5. Differential Correlation Network Analysis of TSG Affected Metabolites

In the differential correlation networks (model vs. sham and TH vs. model, Figure 3C,D) and the scatter plots of corresponding metabolite pairs (Figure 3E), some metabolic changes were clearly observed. Positive correlations of lactate (significantly increased in the model group) with isoleucine, leucine, valine, ascorbate and succinate in model vs. sham differential network, and significant decreases of AMP and ATP in the model group indicated an accelerated glycolysis and energy difficulty. In TSG-treated MCAO rats, lactate was markedly decreased and showed positive correlations with ATP, AMP, ADP and choline in TH vs. the model differential networks, indicating great amelioration of TSG on energy metabolism. Another notable difference between the two differential network lies in the purine metabolism (AMP, ATP, xanthine and inosine), which were located at the center of the TH vs. model network but in the margin of the model vs. sham network. Strong positive correlations (xanthine/ATP, ATP/AMP) dominated the TH vs. model differential network, which were missing in the model vs. sham differential network. These results suggested energy metabolism and purine metabolism are strongly implicated in the neuroprotection of TSG.

### 3.6. DPCPX Abolished the Neuroprotection of TSG in Stroke Rats

**Figure 4:**
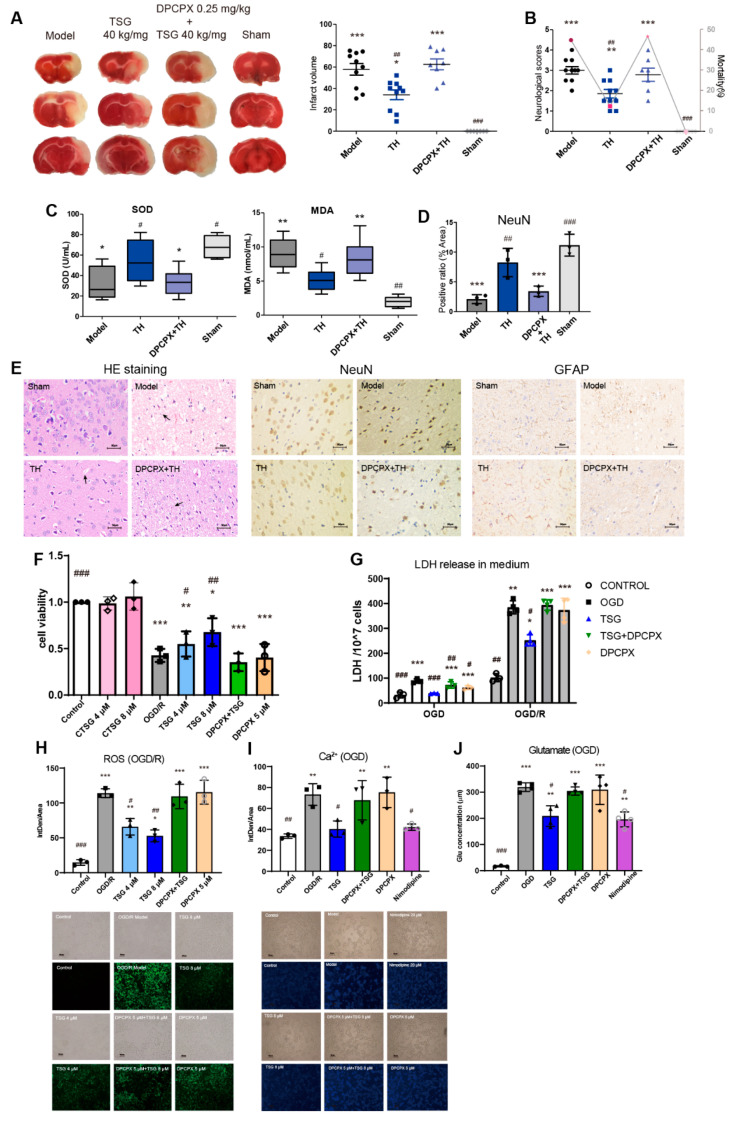
DPCPX abolished the neuroprotection of TSG in vivo and in vitro. (**A**) TTC staining of cerebral slices and cerebral infarct volume percentage (% of hemisphere), *n* ≥ 8. (**B**) Neurobehavioral scores and mortality of rats (*n* ≥ 7). (**C**) Boxplots of biological parameters SOD, MDA (*n* = 4). (**D**) Bar plots for the positive expression ratio of NeuN (*n* = 3). (**E**) HE staining, NeuN and GFAP immunostainings of brain sections from the model, TH, DPCPX + TH and sham groups, *n* = 3. Scale bar represents 50 µm. (**F**) Cell viability of SH-SY5Y cells with different treatment (CTSG: Control + TSG; DPCPX + TSG: 5 µM, 8 µM, respectively) after OGD/R and under normoxic condition was determined by the CCK-8 assay. (**G**) Cellular LDH release were determined after OGD 3 h and OGD/R with treatment of TSG (8 µM), DPCPX (5 µM), DPCPX (5 µM) + TSG (8 µM) combination. (**H**) Intracellular ROS in OGD/R cells was reflected by the fluorescence intensity with DCFH-DA probe (intracellular ROS assay) stained. Representative immunofluorescence images of SH-SY5Y cells are shown. Scale bar represents 100 µm. (**I**) Ca^2+^ influx was determined by immunofluorescence staining assay after OGD. Representative immunofluorescence images of SH-SY5Y cells are shown. Scale bar represents 50 µm. (**J**) Glutamate release in medium was determined by glutamate assay kit after OGD. The mean fluorescence intensity was calculated by Image J software. # *p* < 0.05, ## *p* < 0.01 and ### *p* < 0.001, other groups vs. model/OGD/R group; * *p* < 0.05, ** *p* < 0.01 and *** *p* < 0.001, other groups vs. sham/control group.

The protective effect of TSG (40 mg/kg, s.c.) could be abolished by pretreatment with DPCPX (0.25 mg/kg, s.c.), a specific potent A1R antagonist. With DPCPX pretreatment, the mortality rate, neurobehavioral deficit scores and cerebral infarction volume of MCAO rats were significantly increased after TSG treatment (Figure 4A,B). TSG-enhanced antioxidant capacity was also reversed by DPCPX; similar to the model group, significantly increased cerebral MDA level and decreased SOD activities were observed in the DPCPX + TH group (Figure 4C). The neuron intensity in the DPCPX + TH-treated group was decreased to an extent comparable to the model group, suggesting DPCPX pretreatment induced worsened damage in nerve cells (Figure 4D,E). Moreover, irregular neuroglia arrangement and increased GFAP immunoreactivity in the penumbra were observed in the DPCPX + TH group. These results suggested that A1R might be responsible for the anti-stroke ability of TSG.

### 3.7. TSG-Increased Cell Survival during OGD/R was Abolished by DPCPX

Exposure to OGD impaired cells, as evidenced by the observed morphological changes, reduced cell density and swelling, and cell necrosis, indicative of cell injury or death at 24 h after the insult, and rapid decrease in cell viability (Figure 4F, Supplementary Figure S1C). Treatment with 4/8 μM TSG for 24 h significantly increased cell viability of OGD/R cells, which maintained their morphology quite well as compared with those untreated (Figure 4F). The amount of cell LDH leakage into the culture medium was significantly increased by both OGD and OGD/R (Figure 4G), indicating high membrane permeability due to severe cell membrane damage during OGD/R. In normoxic condition, TSG (4/8 μM) has no significant effects on cell viability and LDH release on cells (Supplementary Figure S1A,B). TSG greatly relieved oxidative stress in cells as evidenced by a notable decrease of intracellular ROS produced by OGD/R (Figure 4H). With the increase of membrane permeability, glutamate release and Ca^2+^ influx increased (Figure 4I,J). TSG significantly decreased LDH and glutamate release into the culture medium and prevented Ca^2+^ influx induced by OGD. The A1R-selective antagonist, DPCPX (5 µM) aggravated cell damage (slight but not significant) and abolished the neuroprotective effects of TSG on OGD/R cells. The positive control nimodipine (20 µM), a calcium channel antagonist, significantly inhibited Ca^2+^ overload and glutamate release.

### 3.8. Docking Results Using AutoDock4 and AutoDock Vina

**Figure 5:**
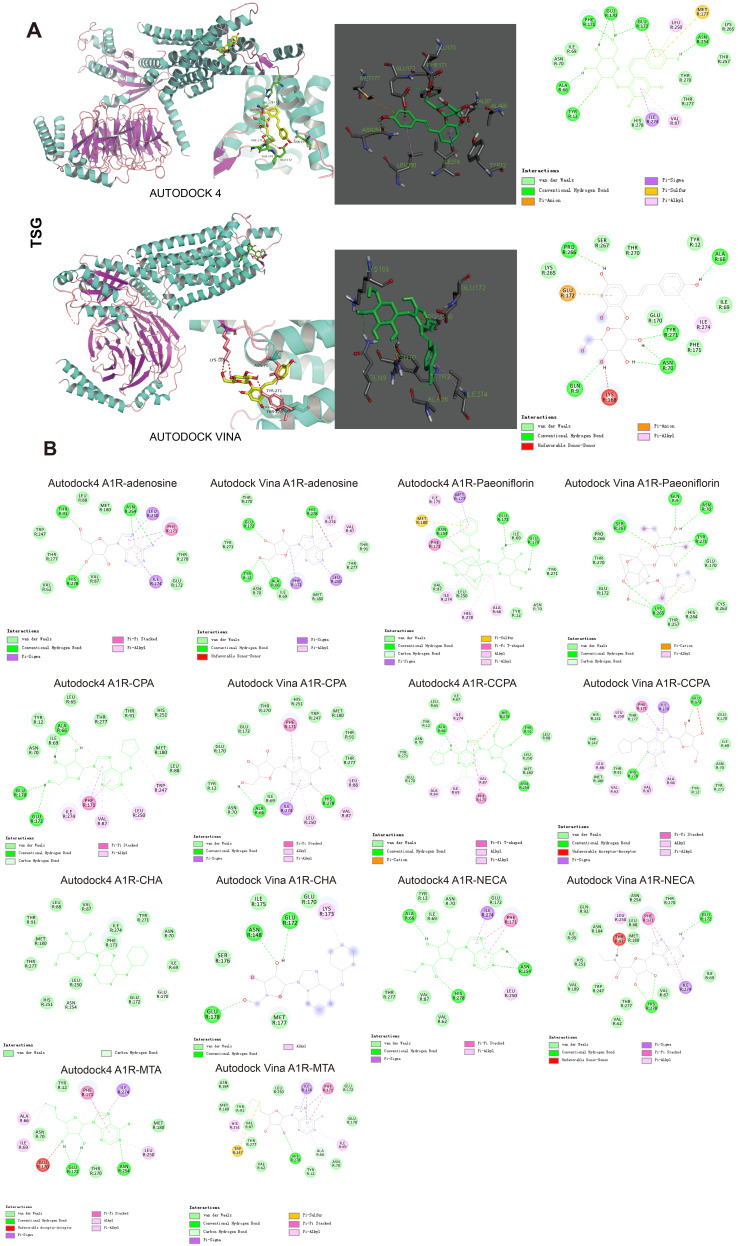
Molecular docking analyses between A1R and its agonists. (**A**) The output of AutoDock4 and AutoDock Vina showed the binding site residues of A1R with the ligand TSG edited in PyMOL/Discovery Studio 4.5 software. TSG (yellow stick format) was docked in the lowest energy conformation inside the binding pocket of A1R (shown in marine and violet). Detailed binding site residues (green color) of A1R protein with the ligand TSG (green stick format) and 2D diagram showed the types of contacts formed between A1R and TSG. The green dotted lines indicated H-bond interactions between A1R and TSG. (**B**) Two-dimensional diagrams showing the types of contacts formed between A1R and its known agonists (adenosine, paeoniflorin, CPA, CCPA, CHA, NECA and MTA) output by AutoDock4 and AutoDock Vina.

Molecular docking was performed to evaluate the binding affinity between A1R and TSG using AutoDock4 and AutoDock vina software. Potent and selective A1R agonists CCPA, CPA, CHA, MTA (derivatives from adenosine), paeoniflorin (an A1R activator with neuroprotective and antidepressant effects [34]) and adenosine (an endogenous A1R receptor agonist) were also docked into A1R for docking accuracy comparison. The best selected poses of the A1R-TSG docked complex were finalized based on binding energy: −7.71 and −7.0 kcal/mol calculated by AutoDock4 and AutoDock vina, respectively, with hydrogen bonds and binding site residues as shown in Figure 5A. Amino acid residues (ALA-66, PHE-171, ASN-254, TYR-12, GLU-170 and GLU-172 for AutoDock4; GLN-9, ASN-70, TYR-271, ALA-66 and PRO-266 for AutoDock vina) were involved in the interactions of TSG with A1R through hydrogen bonding.

Two-dimensional diagrams of the best-selected poses of the known A1R agonists and their binding site residues are shown in Figure 5B, with the corresponding lowest binding energy listed in Table 2. The notable interactions between the endogenous A1R agonist-adenosine and A1R includes π-stacking with PHE-171, hydrogen bonding with ASN-254, HIS-278 and GLU-172, Van der Waals interactions with MET-180, other non-covalent interactions with TRP-247, VAL-87, THR-91 and THR-277. The binding affinity between TSG and A1R was stronger than adenosine, CHA and MTA in terms of binding energy. The hydrogen bond interactions of TSG were similar to those of adenosine (ASN-254, GLU-172, ALA-66, TYR-12), paeoniflorin (ASN-254, GLU-172, TYR-12), CCPA (ALA-66, ASN-254, GLU-172), CHA (ASN-254, GLU-172, GLU-170) and NECA (ALA-66, HIS-278, GLU-172), indicating TSG might also be an A1R agonist.

### 3.9. TSG Bound with A1R and Activated ERK1/2 Phosphorylation

**Figure 6:**
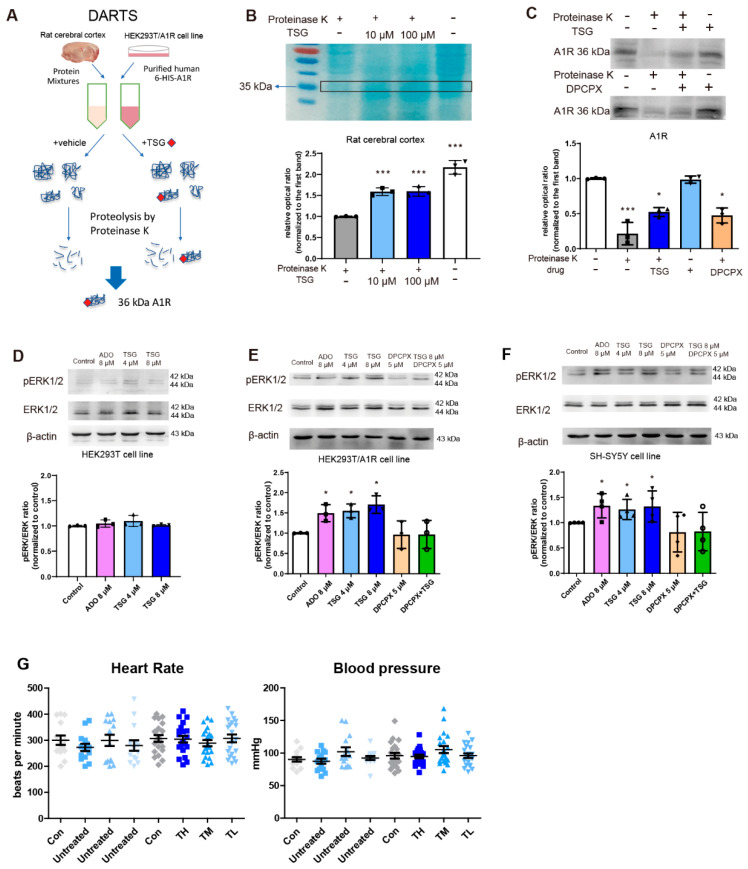
TSG targeted A1R and activated ERK1/2 phosphorylation, a downstream signal of A1R. (**A**) Experimental procedure of DARTS with TSG. (**B**) Coomassie SimplyBlue staining of the cerebral lysates subjected to proteinase K digestion (*n* = 3). (**C**) SDS-PAGE analysis of the cellular lysates subjected to proteinase K digestion with A1R antibody staining (*n* = 3). (**D**) The phosphorylation of ERK1/2 in HEK293T cells without A1R was detected by Western blot after 24 h incubation with adenosine (ADO, 8 µM) and TSG (4 µM and 8 µM). (**E**,**F**) The phosphorylation of ERK1/2 in HEK293T/A1R cells and SH-SY5Y cells was detected by Western blot after 24 h treatment of ADO (8 µM), DPCPX (5 µM) and TSG (4 µM and 8 µM). The gray value was analyzed by ImageJ software. All the values were expressed as mean ± SD, *n* ≥ 3. (**G**) The heart rate and blood pressure of rats 14 days before TSG treatment and 21 days with TSG treatment, respectively. TSG was treated for rats for 21 days with 40, 20 and 10 mg/kg (s.c., twice a day) corresponding to the TH, TM and TL groups. Each point represents the mean value of blood pressure and heart rate of rats each day in the same group. The observed vertical bar represents the mean ± SD of the blood pressure and heart rate at 14 days and 21 days, respectively. * *p* < 0.05, and *** *p* < 0.001, other groups vs. Control group.

In the DARTS experiment, after proteinase K digestion, lysates of TSG-treated brain tissues exhibited much stronger staining intensities than those untreated around 36 kDa protein band, corresponding to the molecular weight of A1R (Figure 6B). To resolve the identity of the protein, A1R was purified and incubated in the presence/absence of TSG incubation; the intensity of A1R bands (denoted by A1R antibody) was significantly higher in the presence of TSG (Figure 6C). Similar findings were observed for DPCPX, a potent A1R antagonist. Protection of A1R from proteinase K digestion by TSG demonstrated its strong bonding to A1R.

The phosphorylation of ERK1/2 (pERK1/2), a downstream signal linked to A1R activation, was examined to test the biological function of A1R with TSG binding. The expression of pERK1/2 was nearly unchanged in untransfected HEK293 cells after the treatment of TSG (4, 8 µM) or adenosine (ADO, 8 µM) (Figure 6D). In contrast, both TSG and adenosine could stimulate pERK1/2 in stably transfected HEK293T/A1R cells and SH-SY5Y cells (Figure 6E,F). DPCPX significantly decreased pERK1/2 and inhibited its upregulation by TSG. On ERK1/2 phosphorylation, TSG functioned similarly to A1R agonist, but conversely to A1R antagonist. These results suggest that TSG bound with A1R as its agonist and the activation of pERK1/2 by TSG was dependent on A1R activation. Unlike classical A1R agonists, TSG had no influence on the blood pressure and heart rate of healthy SD male rats with 21 day’s TSG administration (Figure 6G).

### 3.10. Activation of ERK1/2 Signaling Pathway Contributed to TSG’s Neuroprotection

**Figure 7:**
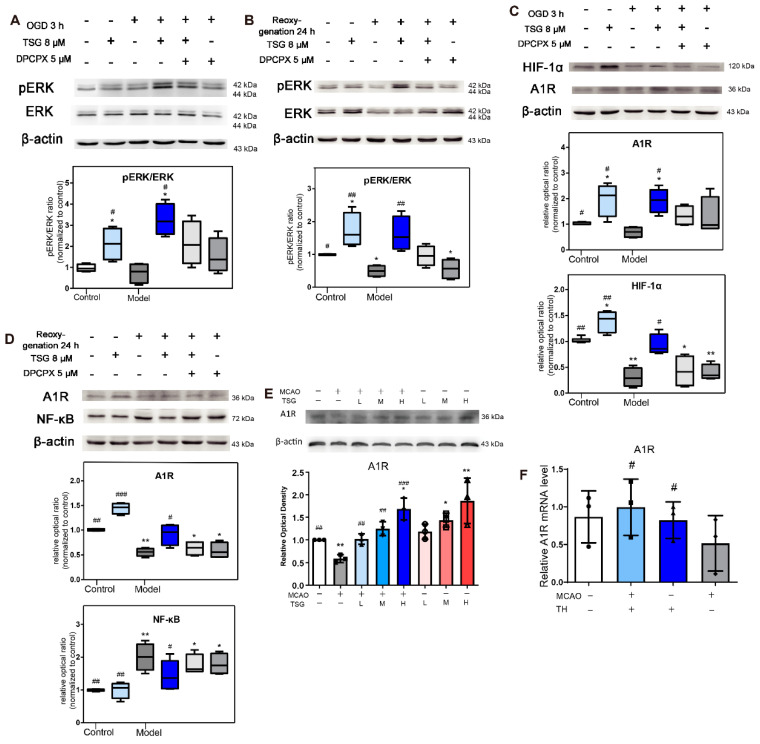
Effects of TSG on the expression of HIF-1α, A1R, NF-κB, ERK1/2 and pERK1/2 in OGD cells. (**A,B**) The expression of ERK1/2 and pERK1/2 in SH-SY5Y cells harvested after OGD 3 h and OGD/R (3 h/24 h) and the relative intensity ratio of pERK/ERK (*n* = 4). (**C**) The expression of A1R, HIF-1α in SH-SY5Y cells harvested after OGD (*n* = 4). (**D**) The expression of A1R, NF-κB in SH-SY5Y cells harvested after OGD/R (*n* = 4). (**E**) The expression of A1R in rats of each group (*n* = 3). (**F**) The gene expression levels of A1R affected by MCAO and TSG in brains by Q-RT-PCR in each group (*n* = 3). The relative intensity was analyzed with ImageJ software and calculated by the ratio relative to the β-actin intensity. # *p* < 0.05, ## *p* < 0.01, and ### *p* < 0.001, other groups vs. the model, OGD or OGD/R groups; * *p* < 0.05, ** *p* < 0.01, other groups vs. the control group.

Activation of the ERK1/2 signaling pathway ameliorated injury in neurons exposed to I/R [35]. Cellular pERK1/2 levels in the case of OGD and OGD/R were significantly lower than those in normoxia cells (Figure 7A,B). TSG (8 µM, 24 h) markedly increased pERK1/2 levels as compared with non-TSG-treated OGD/R cells and in normoxic cells (Supplementary Figure S2A,B). DPCPX (5 µM) pretreatment significantly inhibited ERK1/2 phosphorylation and reversed TSG (8 µM) induced pERK1/2 upregulation in OGD/R cells. DPCPX intervention aggravated OGD-induced cell damage as evidenced by higher extracellular LDH activity and lower cell viability, which indicated that inhibition of A1R and ensuing ERK1/2 pathway activation exacerbated OGD/R-induced cell injury. DPCPX could also eliminate the neuroprotection of TSG through its competitive binding with A1R. Therefore, activation of A1R and its downstream ERK1/2 pathway underlie the neuroprotective effects of TSG on OGD/R damage in SH-SY5Y cells.

### 3.11. TSG Increased A1R Expression

A sufficient A1R level would be extremely important for an A1R agonist to act as a successful stroke therapy. Thus, we studied the changes in A1R expression after MCAO, OGD and OGD/R. TSG increased A1R level in OGD, non-OGD/R and OGD/R-treated cells (Figure 7C,D). The mRNA and protein expressions of A1R were greatly decreased in the MCAO model group, which could be reversed by TSG (Figure 7E,F). Co-administration of DPCPX attenuated TSG upregulation of A1R, thus aggravating OGD/R damage.

### 3.12. HIF-1α and NF-κB Were Involved in the Neuroprotection of TSG

HIF-1α is considered to be the main regulator of angiogenesis and responsor to hypoxia [36]. Though quickly degraded via the ubiquitin-proteasome pathway in normoxia, HIF-1α was stable under hypoxia and regulated the expression of target genes by interaction with coactivators such as cAMP response element-binding protein (CREB) [36]. Different from the previous studies on hypoxia, the level of HIF-1α was obviously decreased in OGD-induced anoxic SH-SY5Y cells. TSG (8 µM) significantly increased HIF-1α levels in both control and OGD cells (Figure 7C, Supplementary Figure S2A,C). The transcription factor NF-κB is a key regulator of hundreds of genes involved in cell survival and inflammation. There is ample evidence that NF-κB is activated in cerebral ischemia [37]. In OGD/R SH-SY5Y cells, the protein levels of NF-κB were significantly increased, which could be inhibited by TSG (Figure 7D). DPCPX blocked the effects of TSG on HIF-1α and NF-κB expressions, demonstrating close associations of HIF-1α and NF-κB with TSG augmented A1R level in OGD-induced anoxia.

## 4. Discussion

A1R, a purinoceptor linked to the downstream cAMP signaling pathway, is one of the predicted targets of TSG with a high score. A1R is highly expressed in the brain and regulates a range of physiological functions [38]. Activation of A1R in the brain could ameliorate ischemic injury in vivo, and hypoxic damage and death in vitro [39,40]. I/R leads to cell death and adenosine release, which activates A1R to protect cells from ischemic damage by reducing the energy demand of the neurons [5]. Upon activation, A1R reduced the energy demands of the neuronal tissue, thus serving as a self-protection strategy to attenuate ischemic damage [5]. However, the extracellular adenosine level is unstable, thus anoxia-induced adenosine increase typically only leads to a temporary ischemic tolerance [41]. This self-protection mechanism was deprived in cerebral ischemia and stroke [42], where both protein and mRNA levels of A1R were decreased [43]. Searching for A1R agonists targeting functional A1R sites present in the penumbra may be an effective therapeutic option for stroke treatment. For example, injection of the A1 agonists cyclohexyladenosine (CHA) improves neurological deficits, protects the CA1 region of the hippocampus and prevents the reduction of A1R in rats or gerbils [44].

In this study, we first investigated the neuroprotective effects of TSG in vivo and in vitro in the absence/presence of DPCPX, a selective potent A1 antagonist. DPCPX intervention almost completely abolished the anti-ischemia ability of TSG, especially the anti-oxidative activity, suggesting that A1R activation was related to the neuroprotection of TSG (Figure 4). Initially, molecular docking was performed to assess protein–ligand binding potential between TSG and several A1R agonists CCPA, CPA, CHA, MTA, NECA, paeoniflorin and adenosine. Crystallographic experiment has confirmed notable interactions between adenosine and A1R orthosteric site residues [45]. TSG bound to A1R with lower binding energy than adenosine, CHA and MTA. Its binding sites, TYR-12, GLU-172, ASN-254, ALA-66, GLU-170, were similar to other A1R agonists, indicative of an A1R agonist nature of TSG (Figure 5). Clinically, use of classical A1 agonists is hampered by undesirable side effects such as sedation, bradycardia and hypotension [46,47]. A1R agonists without hemodynamic effects are more valuable drugs in ischemic stroke treatment [47]. Inspiringly, unlike classical A1R agonists, TSG had no obvious effect on the blood pressure and heart rate of rats (Figure 6G). TSG could protect A1R from proteinase K digestion according to DARTS analysis, which confirmed the binding of TSG with A1R (rat and human sources). In addition, TSG functioned similarly with A1R agonist-adenosine but conversely with A1R antagonist DPCPX in regards to ERK1/2 signaling. The upregulation of pERK1/2 (the downstream signal of A1R activation) by TSG demonstrated itself an agonist of A1R. Apart from activating A1R during I/R, TSG could significantly upregulate the protein and mRNA expression of A1R in both the model and sham rats (Figure 7E,F). TSG treatment could both upregulate and activate A1R: this “one for two” characteristics is favorable for its protection against I/R injury.

Activation of the ERK cascade could inhibit apoptosis and rescue cells from injury following I/R, and help the restoration of memory and learning ability [48,49]. High dosage of TSG potently upregulated ERK1/2 phosphorylation in either non-OGD-treated or OGD/R-treated SH-SY5Y cells, and enhanced the survival of OGD/R cells. DPCPX inhibited TSG induced ERK1/2 phosphorylation, and thus aggravated OGD/R damage. A1R downstream ERK1/2 signals could sense the intracellular AMP/ATP ratio, and thus function as important regulators of energy homeostasis [50]. Activation of the ERK1/2 pathway shuts down non-essential synthetic pathways to decrease energy demand while promoting energy production [50]. Cerebral ischemia begins as an imbalance between reduced energy supply and the high-energy demand of the brain, and thus inevitably has a close relationship with purine metabolism [51]. The cerebral changes of purines AMP, ATP and adenosine levels after MCAO modeling were significantly reversed by TSG treatment. These purines presented strong positive mutual correlations in the TH vs. model differential network. Pathway enrichment analysis performed on metabolites revealed great significance of purine metabolism. After further incorporation of predicted targets, purine metabolism has much greater importance together with the cAMP signaling pathway: A1R and ERK signaling pathways are its two subpathways. TSG treatment expedited energy production and reduced its expenditure, which was favorable to help the brain manage the energy dilemma and survive the harmful I/R.

In vitro, TSG depressed cellular activity and energy consumption, which was partly due to the inhibition of Ca^2+^ influx and glutamate release, just like A1R natural agonist adenosine [52,53]. The relationships between adenosine and its regulation on energy metabolism are not as straightforward as previously thought: Excessive extracellular adenosine release may be lethal and loss of adenosine after a long period of ischemia limits the resynthesis of ATP during reperfusion [54,55], and thus aggravates the severity of brain damage. Interestingly, TSG treatment alleviated the loss of intracellular adenosine in the brain of MCAO rats and significantly restored the decreased contents of ATP, AMP after I/R (Table 1). TSG ameliorated lactate accumulation in the brain of MCAO rats, which, together with the observed positive correlations between lactate and purine metabolites (ADP, AMP and xanthine), demonstrated great improvement on energy metabolism. In contrast, these correlations were missing in the MCAO model group, indicating severe disorder of energy homeostasis in ischemic brains (Figure 3C,D).

TSG is well known for its strong antioxidant and free radical scavenging activities [23]. ROS produced by I/R increases lipid peroxidation, and oxidizes enzymes and proteins. TSG significantly lowered the oxidative stress level and significantly ameliorated I/R-induced increase of amino acids due to protein degradation in I/R brain. ROS could destroy plasma membrane integrity and disturb ion (Ca^2+^, K^+^, Na^+^) homeostasis [56], resulting in cell swelling and necrosis. In the case of Ca^2+^ overload and accumulation in neurons, ROS were produced by the activation of prooxidant pathways [57,58], resulting in a vicious circle. Since A1R activation could inhibit Ca^2+^ overload in OGD/R cells [59], A1R agonists could decrease ROS production while A1R antagonist increased the ROS level [60]. DPCPX pretreatment blocked the antioxidant effects of TSG both in vivo and in vitro, indicating the contribution of A1R to the antioxidant activity of TSG. Moreover, A1R activation also underlies the neuroprotective effects of TSG in mediating other endogenous responses as DPCPX blocked the effects of TSG on HIF-1α, pERK and NF-κB protein level in OGD/R cells. HIF-1α shifts oxidative phosphorylation toward glycolysis under hypoxia/anoxia to inhibit excessive accumulation of ROS [58,61]. A striking decrease of HIF-1α was found following three hours of anoxia (O_2_, 0.1%) in our study, which suggested that HIF-1α is far less stable in anoxia (O_2_, 0.1%) than in hypoxia (O_2_, 1%) [62]. In addition, TSG significantly inhibited OGD/R up-regulation of NF-κB (Figure 7D), which is critically involved in neuronal death [63]. TSG significantly upregulated the protein level of pERK and HIF-1α at dosages of 0.5 and 1 µM, respectively (Figure S2). The influence of an agonist varies with its dosage and receptor function, TSG exerted its efficacy in both normoxic and OGD condition at a wide concentration range (0.5–8 µM), showcasing its great potential for prevention and treatment of stroke.

## 5. Conclusions

**Figure 8:**
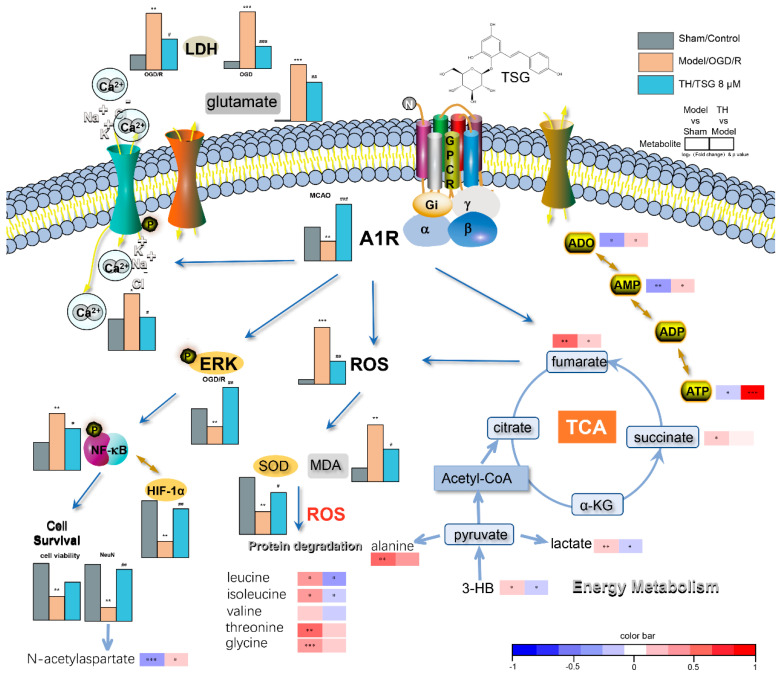
A schematic picture of TSG neuroprotection. TSG exerted its neuroprotective effect against cerebral I/R injury by activating A1R, upregulating its level, and triggering its downstream signaling pathways. TSG reversed the disturbed energy and amino acids metabolism in the brain of MCAO rats, ameliorated oxidative stress in vivo and in vitro. OGD/R induced Ca^2+^ overload and excessive glutamate release, decreased phosphorylation of ERK1/2 and HIF-1α stability and increased NF-κB level, which could be reversed by TSG. α-KG, α-ketoglutarate; TCA cycle, tricarboxylic acid cycle. # *p* < 0.05, ## *p* < 0.01 and ### *p* < 0.001, other groups vs. model/OGD/R group; * *p* < 0.05, ** *p* < 0.01 and *** *p* < 0.001, other groups vs. sham/control group.

A1R activation by endogenous adenosine during I/R exerted neuroprotection against I/R injuries. However, such an innate protective pathway is temporal and transient. In our studies, acting as an A1R agonist, TSG activated and upregulated A1R, thus exerting multifaceted neuroprotective abilities against I/R injury (Figure 8). TSG activated A1R and its downstream effector pERK1/2 that enhanced energy production and reduced energy expenditure, and thus helped the brain manage the energy dilemma and survive the harmful I/R, contributing to the anti-stroke effects of TSG. The A1R activation also underlay the antioxidative activity of TSG. In addition, few cardiovascular side effects add further value to TSG for its development for stroke treatment.

## Figures and Tables

**Table 1:** Fold changes of brain metabolites between groups and the associated *p*-Values.

Brain Metabolites	Model vs. Sham		TH vs. Model		TM vs. Model		TL vs. Model	
	log_2_ (FC)	*p*	log_2_ (FC)	*p*	log_2_ (FC)	*p*	log_2_ (FC)	*p*
Isoleucine	0.41	*	−0.12	*	−0.18	*	−0.28	*
Leucine	0.30	*	−0.34	*	−0.29	*	−0.43	*
Valine	0.20		−0.11		−0.22		−0.39	
3-hydroxybutyrate	0.24	*	−0.10	*	0.02		−0.37	*
2-hydroxyisobutyrate	0.78	***	0.22		0.28	*	0.09	
Lactate	0.24	**	−0.14	*	−0.28	*	−0.29	*
Alanine	0.63	**	0.39		0.31		0.12	
Lysine	0.05		−0.09		−0.03		−0.24	
4-Aminobutyrate	−0.18	*	0.25	***	0.19	**	0.22	***
N-acetyl aspartate	−0.37	***	0.14	*	−0.13		0.04	
Glutamate	0.07		−0.05		0.09	*	0.01	
Succinate	0.15	*	0.09		−0.04		0.39	*
Glutamine	0.11		−0.12		−0.03		−0.11	
Aspartate	−0.18	**	0.14	*	0.07		−0.06	
Trimethylamine	-0.09		−0.32	***	−0.28	**	−0.28	*
Creatine	−0.18	*	0.05		−0.10	*	−0.14	
Choline	0.19	*	−0.06		−0.05		−0.12	
Phosphocholine	−0.01		0.09		0.16		−0.07	
Taurine	0.00		0.02		0.13		0.09	
Myo-inositol	0.04		0.01		0.05		0.10	
Glycine	0.48	***	0.21		0.30		0.18	
Threonine	0.52	**	0.21		0.17		0.21	
Ascorbate	−0.20	***	0.15		0.44	*	0.01	
Adenosine	−0.50	*	0.17	*	0.10	*	0.05	
Inosine	0.06		−0.29	*	−0.47	*	−0.37	*
Uridine	−0.02		0.08		−0.15		−0.09	
Uracil	0.22		0.19		0.13		0.20	
AMP	−0.38	**	0.20	*	0.56	*	0.52	*
ADP	0.09		0.27		0.39		0.01	
ATP	−0.21	*	1.33	***	1.34	**	0.97	*
Fumarate	0.67	**	0.24	*	0.29	*	0.09	
Tyrosine	−0.10		−0.06		0.03		0.02	
Phenylalanine	0.25	*	−0.07		−0.13		−0.05	
Niacinamide	−0.09		0.05		−0.13		0.05	
Histidine	0.01		0.09		−0.05		0.97	
Xanthine	−0.29	**	−0.06		−0.08		−0.19	
Oxypurinol	0.38		0.09		0.33	*	0.27	
Formate	0.40	*	0.73	**	0.82	**	0.92	***

AMP, adenosine monophosphate; ADP, adenosine diphosphate; ATP, adenosine triphosphate. FC: Color coded according to the fold-change value; color coded according to log_2_ (FC), red represents increased and blue represents decreased concentrations of metabolites. Color bar. 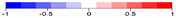. *p*-Values corrected by Benjamini–Hochberg methods were calculated based on a parametric Student’s *t* test or a nonparametric Mann–Whitney test (dependent on the conformity to normal distribution). * *p* < 0.05, ** *p* < 0.01 and *** *p* < 0.001.

**Table 2:** Docking results with human A1R (PDB ID: 6D9H).

Drug	Binding Energy Calculated by Autodock4 (kcal/mol)	Binding Energy Calculated by Autodock Vina (kcal/mol)
CCPA	−7.24	−7.9
CPA	−7.1	−8.1
CHA	−5.1	−5.4
MTA	−5.62	−6.4
NECA	−5.51	−7.6
Paeoniflorin	−7.39	−6.9
TSG	−7.71	−7.0
Adenosine	−6.49	−6.1

CCPA, 2-Chloro-N6-cyclopentyladenosine; CPA, N6-Cyclopentyladenosine; CHA, N6-Cyclohexyladenosine; MTA, 5′-Deoxy-5′-methylthioadenosine; NECA, 1-(6-Amino-9H-purin-9-yl)-1-deoxy-N-ethyl-β-D-ribofuranuronamide.

## Data Availability

Metabolomics data have been deposited in the EMBL-EBI MetaboLights database under the accession code MTBLS2369 (https://www.ebi.ac.uk/metabolights/index, accessed on 1 February 2021). Raw image of all blots with replications in Figure 6 and Figure 7 and Supplementary Figures S1 and S2 are provided as a supportive data in supplementary materials; each image is named of the appropriate panel of the main figure. All data included in the manuscript are available from the corresponding author on reasonable request.

## Supplementary Materials

The following are available online at https://www.mdpi.com/article/10.3390/antiox10071112/s1, Figure S1: The effects of TSG on SH-SY5Y cells in normoxic conditions, Figure S2: The effects of TSG at different concentrations on the protein level of pERK and HIF-1α in normoxic conditions, Table S1: ^1^H NMR assignment of metabolites in rats’ brain, Table S2: The prediction targets of TSG on stroke.
