# Supplementary material for: Activation of Adenosine A1 Receptor in Ischemic Stroke: Neuroprotection by Tetrahydroxy Stilbene Glycoside as an Agonist
Source: Antioxidants (Basel). 2021 Jul 12;10(7):1112. doi: 10.3390/antiox10071112 (PMC8301086; doi:10.3390/antiox10071112)
Supplement: Supplementary file 1 [file antioxidants-10-01112-s001.zip › antioxidants-1293596-supplementary.pdf]

antioxidants

Supplementary Material: Activation of Adenosine A1 Receptor in Ischemic Stroke: Neuroprotection by Tetrahydroxy Stilbene Glycoside as an Agonist

Lingyu Ruan, Guanghui Li, Wenlong Zhao, Huihui Meng, Qi Zheng and Junsong Wang *

Center for Molecular Metabolism, Nanjing University of Science and Technology, 200 Xiao Ling Wei Street, Nanjing 210094, China; ruanlingyuamy@njust.edu.cn (L.R); lguanghui12@njust.edu.cn (G.L); 217102010089@njust.edu.cn (W.Z); menghuihui2014@njust.edu.cn (H.M); zhengqi@njust.edu.cn (Q.Z.)
* Correspondence: wangjunsong@njust.edu.cn; Tel./Fax: +86-139-1397-6393

Supportive data

Raw images of all blots in Figure 6, 7 and supplementary Figure S1, S2 are provided as a supportive data in supplement; each image is named of the appropriate panel of the main figure. Images of repeated experiments were also included and marked with word R (short for Repeat) in the name suffix.

Supplemental Tables

Table S1. ^{1}H NMR assignment of metabolites in rats' brain.

No.	Metabolites	Assignments	Chemical shifts(ppm)
1	Isoleucine	δCH$_3$, γCH$_3$	0.94 (t), 1.015 (d)
2	Leucine	δCH$_3$	0.97 (dd)
3	Valine	γCH$_3$, γCH$_3$	0.99 (d),1.05 (d)
4	3-Hydroxybutyrate	γCH$_3$	1.21(d)
5	2-Hydroxyisobutyrate	CH$_3$	1.32(s)
6	Lactate	CH$_3$, CH	1.34 (d), 4.12 (q)
7	Alanine	βCH$_3$	1.49 (d)
8	Lysine	δCH2	1.74(m)
9	4-Aminobutyrate	αCH2, βCH2, γCH2	1.91(m), 2.3(t), 3.02(t)
10	N-acetyl aspartate	CH$_3$, CH$_2$	2.02 (s), 2.51 (dd), 2.7 (dd)
11	Glutamate	βCH$_2$, γCH$_2$	2.08(m), 2.36(m)
12	Succinate	CH$_2$	2.41(s)
13	Glutamine	βCH$_2$, γCH$_2$	2.46(m)
14	Aspartate	βCH$_2$, α-CH	2.67(m), 2.82(m)
15	Trimethylamine	CH$_3$	2.88(s)
16	Creatine	CH$_2$, CH$_3$	3.04 (s), 3.93 (s)
17	Choline	N(CH$_3$)$_3$	3.21 (s)
18	Phosphocholine	N-CH$_2$	3.218 (s)
19	Taurine	NH$_2$-CH$_2$, SO$_3$-CH$_2$	3.27 (t),3.43 (t)
20	Myo-inositol	CH	3.52 (dd), 3.61 (t), 4.1 (s)
21	Glycine	CH$_2$	3.57(s)
22	Threonine	C$_2$H	3.585(d)
23	Ascorbate	CH	4.52(d)
24	Adenosine	CH-OH, N=CH-N	6.09(d), 8.25 (s), 8.34 (s)
25	Inosine	O-CH-N, N-CH=N CH=N	6.10 (d), 8.23 (s), 8.34 (s)
26	Uridine	H$_5$, H$_6$, H$_1$'	5.9 (d), 7.9 (d)
27	Uracil	CH=CH-N	5.79(d), 7.54(d)
28	AMP	N=CH-N, N=CH-N	8.23 (s), 8.6 (s)
29	ADP	2'-CH	8.585(s)
30	ATP	2'-CH	8.535(s)
31	Fumarate	CH=CH	6.52(s)

32	Tyrosine	H3/H5, C5H/C6H	6.90 (d),7.20 (d)
33	Phenylalanine	$CH\text{-}NH_2$	7.38(m)
34	Niacinamide	$H_2/H_4/H_5/H_6$	7.58 (dd)
35	Histidine	2'-CH	7.915 (s)
36	Xanthine	NH=CH-N	7.945(s)
37	Oxypurinol	CH	8.245(s)
38	Formate	CH	8.465 (s)

s, singlet; d, doublet; t, triplet; q, quartet; m, multiple; dd, doublet of doublets, dt, doublet of triplets.

Table S2. The prediction targets of TSG on stroke.

Target Name	Organism	id	Score	GENE name
Tyrosinase	Homo sapiens	CHEMBL1973	100	TYR
Aldose reductase	Rattus norvegicus	CHEMBL2622	100	Akr1b7
Sodium/glucose cotransporter 2	Homo sapiens	CHEMBL3884	100	SLC5A2
Coagulation factor III	Homo sapiens	CHEMBL4081	100	F3
Carbonic anhydrase IV	Homo sapiens	CHEMBL3729	100	CA4
Low affinity sodium-glucose cotransporter	Homo sapiens	CHEMBL1770047	100	SLC5A2
Carbonic anhydrase XIV	Homo sapiens	CHEMBL3510	100	CA14
Glycogen phosphorylase, muscle form	Oryctolagus cuniculus	CHEMBL4696	100	PYGM
Carbonic anhydrase VII	Homo sapiens	CHEMBL2326	100	CA7
Maltase-glucoamylase	Homo sapiens	CHEMBL2074	100	MGAM
Carbonic anhydrase XII	Homo sapiens	CHEMBL3242	100	CA12
Dual specificity tyrosine-phosphorylation-regulated kinase 1A	Rattus norvegicus	CHEMBL5508	100	DYRK1A
Glyceraldehyde-3-phosphate dehydrogenase liver	Homo sapiens	CHEMBL2284	100	GAPDH
Acidic alpha-glucosidase	Rattus norvegicus	CHEMBL3513	100	Gaa
Intestinal alkaline phosphatase	Mus musculus	CHEMBL3151	100	Iap
Cytochrome P450 1B1	Homo sapiens	CHEMBL4878	100	CYP1B1
Carbonic anhydrase III	Homo sapiens	CHEMBL2885	100	CA3
Cytochrome P450 1A1	Homo sapiens	CHEMBL2231	100	CYP1A1
Nuclear factor NF-kappa-B p105 subunit	Homo sapiens	CHEMBL3251	100	NFKB1
Carbonic anhydrase VA	Homo sapiens	CHEMBL4789	99.99	CA5A
Carbonic anhydrase IX	Homo sapiens	CHEMBL3594	99.98	CA9
DNA- (apurinic or apyrimidinic site) lyase	Homo sapiens	CHEMBL5619	99.97	OGG1
Carbonic anhydrase XIII	Homo sapiens	CHEMBL3912	99.96	CA13
DNA (cytosine-5)-methyltransferase 1	Homo sapiens	CHEMBL1993	99.62	DNMT1
6-O-methylguanine-DNA methyltransferase	Homo sapiens	CHEMBL2864	99.39	MGMT
Bloom syndrome protein	Homo sapiens	CHEMBL1293237	98.13	BLM
Sucrase-isomaltase	Homo sapiens	CHEMBL2748	97.57	SI
Adenosine A1 receptor	Rattus norvegicus	CHEMBL318	96.59	Adora1
Beta amyloid A4 protein	Homo sapiens	CHEMBL2487	95.19	APP
Carbonic anhydrase VI	Homo sapiens	CHEMBL3025	94.26	CA6
Carbonic anhydrase II	Homo sapiens	CHEMBL205	91.27	CA2
Inosine-5'-monophosphate dehydrogenase 1	Homo sapiens	CHEMBL1822	83.29	IMPDH1
Carbonic anhydrase I	Homo sapiens	CHEMBL261	82.8	CA1
Equilibrative nucleoside transporter 1	Homo sapiens	CHEMBL1997	82.67	SLC29A1
Tubulin beta-1 chain	Homo sapiens	CHEMBL1915	81.1	TUBB1

Macrophage migration inhibitory factor	Homo sapiens	CHEMBL2085	74.94	MIF
Alpha-L-fucosidase I	Homo sapiens	CHEMBL4176	63.53	FUCA1
Transthyretin	Homo sapiens	CHEMBL3194	55.82	TTR
DNA topoisomerase I	Homo sapiens	CHEMBL1781	55.53	TOP1
Solute carrier organic anion transporter family member 1B3	Homo sapiens	CHEMBL1743121	45.16	SLCO1B3
Solute carrier organic anion transporter family member 1B1	Homo sapiens	CHEMBL1697668	41.62	SLCO1B1
Nuclear factor NF-kappa-B p65 subunit	Homo sapiens	CHEMBL5533	35.63	RELA
Ribosomal protein S6 kinase alpha 3	Homo sapiens	CHEMBL2345	25.91	RPS6KA3
Purinergic receptor P2Y14	Homo sapiens	CHEMBL4518	24.95	P2RY14
Sodium/glucose cotransporter 1	Homo sapiens	CHEMBL4979	24.35	SLC5A1

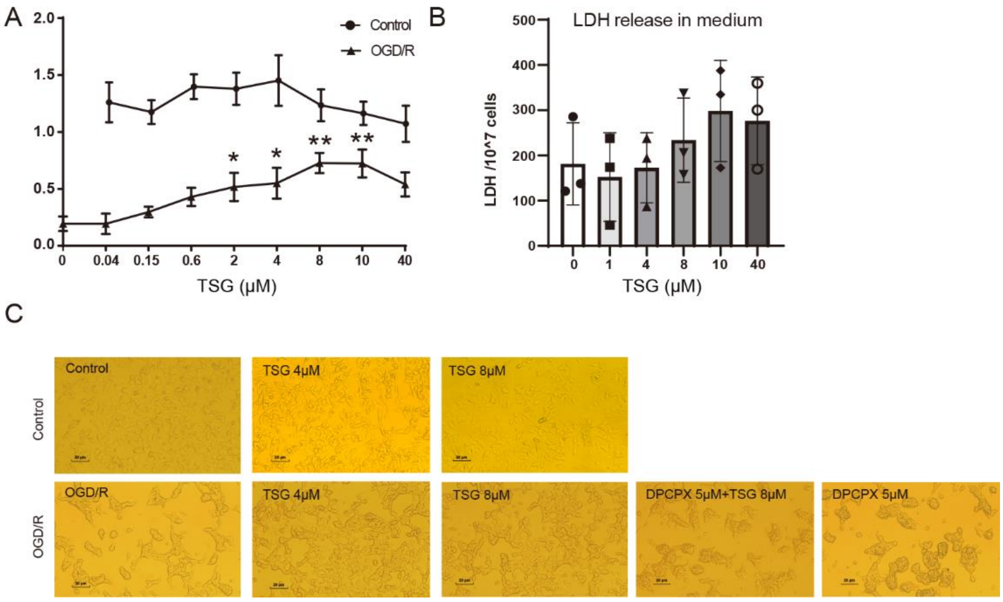

Figure S1. The effects of TSG on SH-SY5Y cells in normoxic conditions. (A) The changes in cell viability with the different concentrations of TSG (24 h) in normoxic conditions/after OGD/R. (B) The changes in LDH release with the different concentrations of TSG (24 h) in normoxic conditions. (C) The changes of cell morphology with 4 and 8 μM TSG (24 h) in normoxic conditions/after OGD/R. * $p < 0.05$, ** $p < 0.01$ and *** $p < 0.001$, other groups versus Model group.

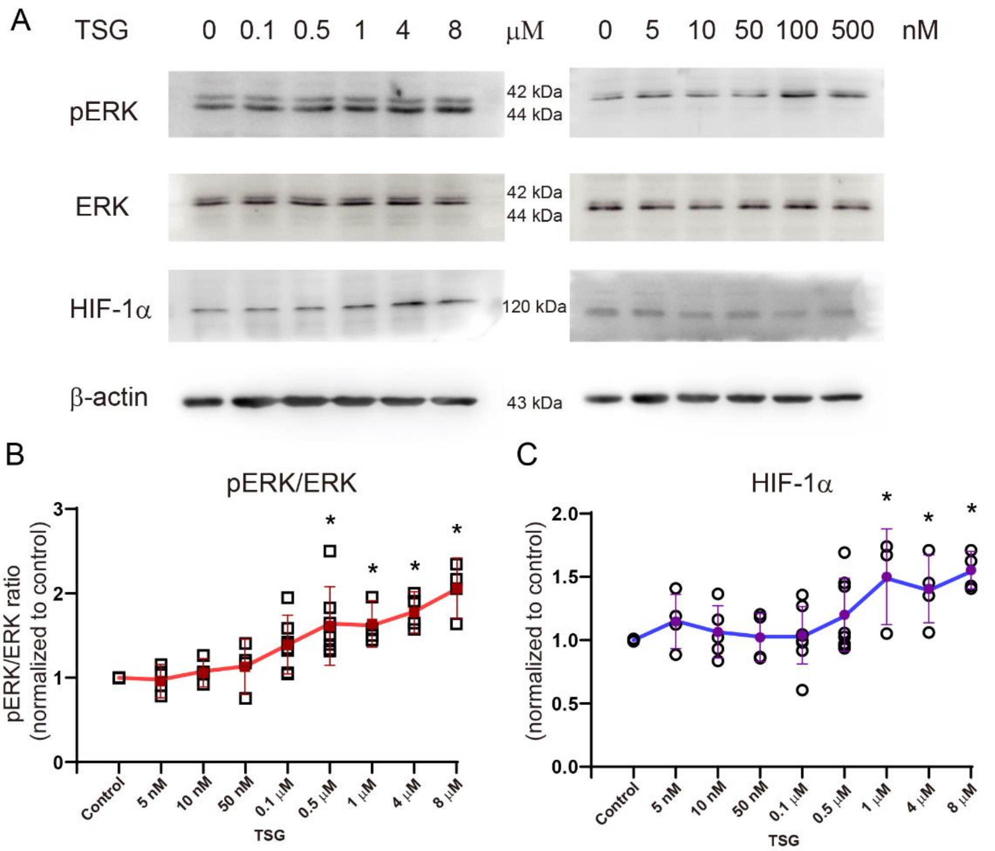

Figure S2. The effects of TSG at different concentrations on the protein level of pERK and HIF-1α in normoxic conditions. (A) The changes of pERK, ERK, HIF-1α and β-actin protein levels in SH-SY5Y cells after incubated with different concentrations of TSG for 24 h in normoxic conditions were tested by western blot assay. (B) The relative intensity was analyzed with Image J software and calculated by the ratio relative to the ERK/β-actin intensity. * $p < 0.05$, ** $p < 0.01$ and *** $p < 0.001$, other groups versus Control group.
